# Contrasting levels of absorption of intense femtosecond laser pulses by solids

**DOI:** 10.1038/srep17870

**Published:** 2015-12-09

**Authors:** Prashant Kumar Singh, Y. Q. Cui, Amitava Adak, Amit D. Lad, Gourab Chatterjee, P. Brijesh, Z. M. Sheng, G. Ravindra Kumar

**Affiliations:** 1 Tata Institute of Fundamental Research, 1 Homi Bhabha Road, Mumbai 400005, India; 2 Beijing National Laboratory of Condensed Matter Physics, Institute of Physics, Chinese Academy of Science, Beijing 100190, China; 3 UM-DAE Centre for Excellence in Basic Sciences, Mumbai 400098, India; 4 SUPA, Department of Physics, University of Strathclyde, Glasgow G4 0NG, UK; 5 Key Laboratory for Laser Plasmas (Ministry of Education), Department of Physics and Astronomy, Shanghai Jiao Tong University, Shanghai 200240, China; 6 Collaborative Innovation Center of IFSA, Shanghai Jiao Tong University, Shanghai 200240, China

## Abstract

The absorption of ultraintense, femtosecond laser pulses by a solid unleashes relativistic electrons, thereby creating a regime of relativistic optics. This has enabled exciting applications of relativistic particle beams and coherent X-ray radiation, and fundamental leaps in high energy density science and laboratory astrophysics. Obviously, central to these possibilities lies the basic problem of understanding and if possible, manipulating laser absorption. Surprisingly, the absorption of intense light largely remains an open question, despite the extensive variations in target and laser pulse structures. Moreover, there are only few experimental measurements of laser absorption carried out under very limited parameter ranges. Here we present an extensive investigation of absorption of intense 30 femtosecond laser pulses by solid metal targets. The study, performed under varying laser intensity and contrast ratio over four orders of magnitude, reveals a significant and non-intuitive dependence on these parameters. For contrast ratio of 10^−9^ and intensity of 2 × 10^19^ W cm^−2^, three observations are revealed: preferential acceleration of electrons along the laser axis, a ponderomotive scaling of electron temperature, and red shifting of emitted second-harmonic. These point towards the role of **J** × **B** absorption mechanism at relativistic intensity. The experimental results are supported by particle-in-cell simulations.

The creation and probing of extreme states of matter is an exciting frontier in science. Among the many possible extremes, those related to high temperature coupled with high density are particularly important for understanding much of the universe, besides being of great value in inertial fusion^1^, particle acceleration^2^, high brightness radiation sources^3^,^4^, plasma processing and many other subjects. Since the advent of the femtosecond, high intensity laser source^5^, there has been tremendous interest in such high energy density science^6^. The development of the Ti-sapphire laser with pulse durations in the tens of femtosecond scale has provoked further questions on the ultrafast dynamics of the generated plasma and how light gets coupled in short scale length of plasma density with *L* < λ, where, 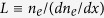, 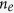 is electron density and λ is laser wavelength. The reality however has been somewhat more complicated because the femtosecond pulses are often preceded by much longer pre-pulses, which are intense enough to produce preplasmas in front of a solid target^7^. It is therefore clear that the intensity contrast between the peak values and the longer duration lower intensity portions is an important parameter. Progress in the last decade has made high contrast, ‘cleaner’ femtosecond pulses available in the lab. It is therefore surprising to note that we still do not have much experimental information on the basic process of absorption of such high contrast laser pulses by even simple solids.

A couple of notable steps in this direction are due to Ping *et al.*^8^ and Pirozhkov *et al.*^9^. The former measured light absorption of ultrahigh intensity (up to 10^20^ W cm^−2^, moderately high contrast (10^−8^), 150 fs, 800 nm laser pulses by micron scale Al foils and 400 micron silica plates and indicated absorption levels as high as 80% at the highest intensities used. The latter obtained data from 50 fs duration and somewhat higher contrast (10^−9^) pulses and came to the opposite conclusion of low level of absorption (<30%) at the highest intensities. Recent models and simulations have proposed novel absorption mechanisms based on the standing wave fields^10^. Moreover, a fully relativistic analytical model presented by Haines *et al.*^11^, based on energy and momentum conservation, predicts light absorption as high as 80%–90% for intensity larger than 10^19^ W cm^−2^. Similarly, another study by Levy *et al.*^12^ highlights the role of hot electrons and hole punching ions in determining the level of light absorption in ultraintense laser-overdense plasma interactions. A very recent theoretical study^13^ has created further excitement by placing bounds on the absorption of the 10^22^ W cm^−2^ intensity pulses by matter. What then is the real illustration of intense, high contrast laser pulse absorption by simple solids like aluminium? In this article we investigate this important, fundamental question by a comprehensive experimental and simulation strategy. The experimental parameter space explored here can be divided into three categories - (a) we vary our laser intensity by four orders of magnitude (10^15^ −10^19^ W cm^−2^), (b) laser intensity contrast by four orders of magnitude (10^−5^ −10^−9^) and (c) use four independent, well established diagnostics (pump reflectivity, angular distribution and energy spectra of fast electrons and Doppler spectrometry of harmonics) to get verifiable and reliable data. Our absorption measurements are sampled over several tens of laser shots at each point in each run and several runs spread over months. We then reproduce this full suite of signals in two-dimensional particle-in-cell simulations. The major results are as follows: (a) a clear trend of increasing absorption with intensity, (b) high absorption for low contrast (10^−5^) and high contrast (10^−9^) pulses. Surprisingly, we observe that at intermediate levels of contrast (10^−7^), there is a drop in absorption, which implies that ‘any’ increase in contrast does not necessarily ensure high absorption.

## Results

### Contrast dependent laser absorption

**Figure 1:**
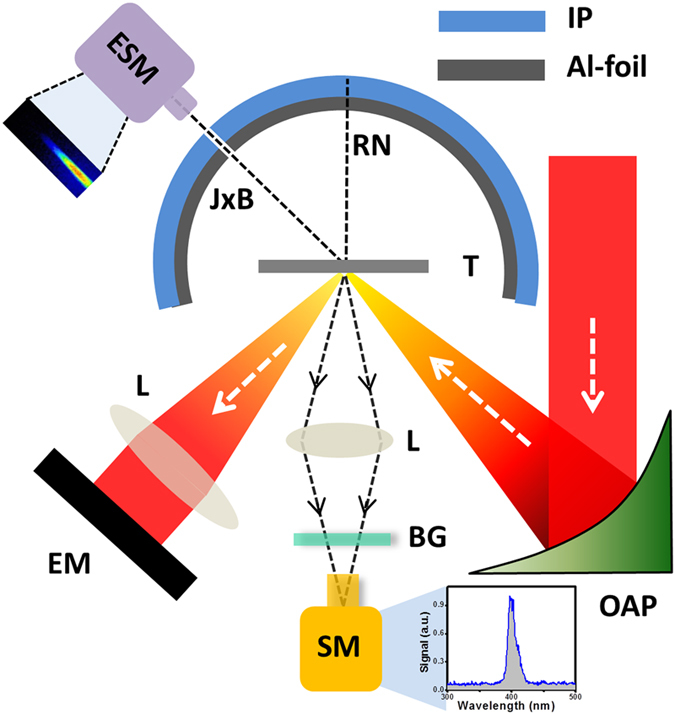
Schematic of experimental set up. An infrared, 30 fs laser pulse, focused by an off-axis-parabola (OAP) excites the target (T) at 45° of incidence. The reflected pulse is fed into a pyroelectric energy meter (EM) after getting collimated by a lens (L). Front side harmonic emission is captured by a spectrometer (SM). An image plate (IP), cover with Al foil, records angular distribution of fast electrons. The energy spectra of fast electron along the laser axis (**J × B**) is measured by an electron spectrometer (ESM). BG: Schott BG-39 filter, RN: rear normal.

**Figure 2:**
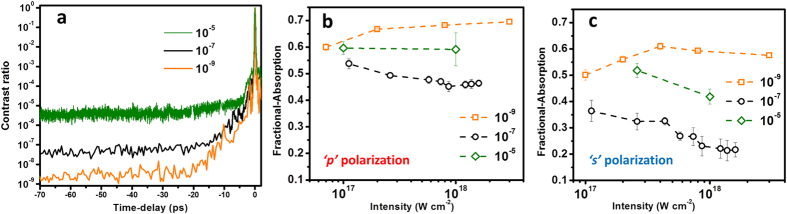
Intensity contrast dependent laser absorption. (**a**) third-order cross-correlator trace of femtosecond laser pulse at three conditions of intensity contrast of 10^−5^, 10^−7^ and 10^−9^. Contrast dependent laser absorption for (**b**) ‘*p*’ polarization and (**c**) ‘*s*’ polarization.

The preplasma scale length is largely dependent on the intensity contrast ratio of a laser pulse, which in turn can significantly control the absorption processes. In order to see the effect of contrast on the absorption, we carry out absorption measurements under three conditions of intensity contrast—namely, 10^−5^, 10^−7^ and 10^−9^. The detailed schematic of the experimental set up is shown in the Fig. 1. Figure 2(a) shows the third order cross correlator scan of the femtosecond laser pulse. The prepulse level is shown up to 70 ps before the femtosecond peak. The pedestal levels at each contrast are separated by nearly two orders of magnitude. Increase in the pedestal level amplifies the preplasma scale length and thereby alters the laser plasma interaction conditions. Figure 2(b,c) show a comparison of absorption measurements over the input intensity range of 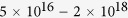 W cm^−2^ for ‘*p*’ and ‘*s*’ polarized light respectively, under the intensity contrast conditions of 10^−5^, 10^−7^ and 10^−9^. The fractional absorption is estimated from 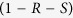, where *R* is the measured reflectivity and *S* is the scattering loss (measured to be less than 5% for 10^−5^ contrast and negligible for higher contrast experiments).

The absorption trend at a contrast of 10^−9^ is quite opposite to that observed at 10^−7^. At 10^−7^ contrast, the absorption across the intensity range is at lower level and further reduces with increasing the intensity. However, further decrease in the intensity contrast to 10^−5^ leads to increase in the fractional absorption for both ‘*p*’ and ‘*s*’ polarization. This reversal is typically attributed to the presence of long scale length preplasma due to low intensity contrast.

### Intensity dependent laser absorption

**Figure 3:**
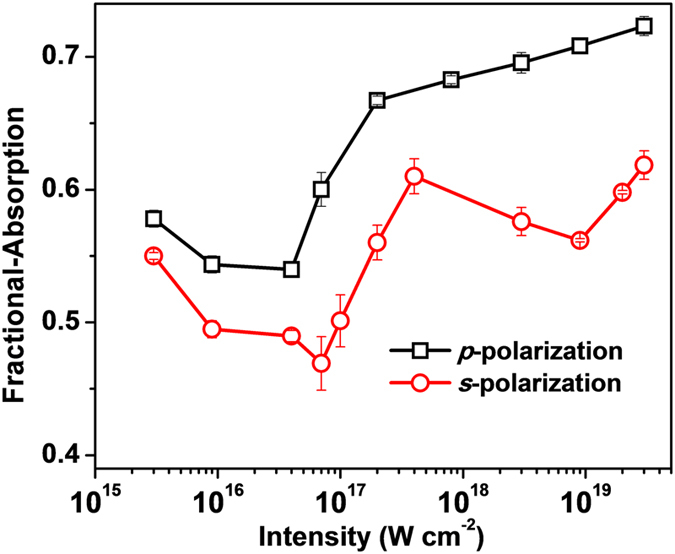
Intensity dependent absorption of high contrast laser. Fractional absorption measurements of ‘*p*’ and ‘*s*’ polarized, 30 fs laser pulse (10^−9^ contrast) for an intensity scan over four orders of magnitude (10^15^–10^19^ W cm^−2^).

Figure 3 shows absorption measurement performed over nearly four orders of intensity scan for ‘*p*’ and ‘*s*’ polarized light with 10^−9^ laser contrast. The fractional absorption for ‘*p*’ polarized light decreases from 57% to a minimum of 53% at the intensity of 4 × 10^16^ W cm‘*p*^−2^. However, for higher intensities the absorption shows a consistent rising trend and reaches 72% at the maximum intensity of 2 × 10^19^ W cm^−2^. The absorption data for ‘*s*’ polarized light also shows similar trend, except in the vicinity of (*I* ~ 10^19^ W cm^−2^ ), where a local dip is observed. Moreover, with respect to ‘*p*’ polarized light, the absorption level for ‘*s*’ polarized light is lower by nearly 10% during most of the intensity scan.

### Angular distribution of fast electrons

**Figure 4:**
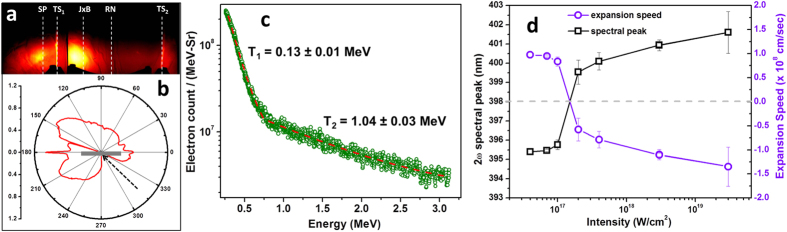
Signature of ponderomotive force. Angular distribution and energy spectra measurements of fast electrons for ‘*p*’ polarized light at intensity of 2 × 10^19^ W cm^−2^. (**a**) The angular distribution of fast electrons recorded in the image plate. SP: specular, TS: target surface; **J** × **B**: laser axis, RN: rear normal. (**b**) Polar plot of the electron flux. The black arrow and grey slab indicates laser direction and target orientation, respectively. (**c**) Energy spectra of fast electrons measured along the **J × B** direction. *T*_1_ and *T*_2_ indicate the fitted temperature of fast electrons. (**d**) Intensity dependent peak of the emitted second harmonic for ‘*s*’ polarized light. The estimated velocity of the critical surface estimated from the Doppler shift is also shown in the same figure. The dotted horizontal line is the location of second harmonic peak estimated from the spectra of the fundamental laser pulse.

The angular distribution of fast electrons is a crucial diagnostic in the determination of the dominance of operating absorption mechanism. In order to understand the absorption mechanisms operating in the high-contrast (10^−9^) experiment, we present results of angular distribution of laser driven fast electrons. The angular distribution of fast electrons in resonance absorption and vacuum heating is known to peak along the target normal, whereas the **J** × **B** mechanism accelerates electrons along the laser axis^7^. Figure 4(a,b) show the angular distribution of fast electrons for ‘*p*’ polarized laser at peak intensity of 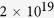 W cm^−2^ with 10^−9^ laser contrast (set up shown in Fig. 1). The angular distribution trace of electrons, recorded in the image plate, is shown in Fig. 4(a). To get a clear picture of the directionality of the fast electrons, we have plotted the angular distribution of electrons in Fig. 4(b). At the front side of the target, a very prominent blob indicates that electrons are accelerated along the laser specular direction, similar to the observation of Habara *et al.*^14^, where in the absence of an external prepulse, the electrons are preferentially accelerated along the direction of the specular reflection of the laser; however in the presence of a preplasma, the electrons are confined along the target surface due to the formation of strong static magnetic fields. Similarly, a very sharp peak is observed along the 180° axis, indicating surface acceleration of electrons^15^. On the opposite side, (0°) a very small peak is observed which implies that the surface acceleration is mostly along the laser specular direction^15^. More interestingly, at the target rear, there is dominance of electrons accelerated along the laser axis (135°) as opposed to target rear normal. In agreement with other experiments^8^ and simulations^16^, this confirms that at relativistic intensity, the **J** × **B** mechanism is much stronger than the resonance absorption or vacuum heating.

### Temperature of fast electrons

Furthermore, the temperature of fast electrons accelerated due to ponderomotive force (**J** × **B** heating) can be given as^17^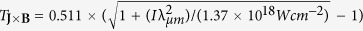. To see the dominance of ponderomotive force, we carried out measurements of electron kinetic energy spectra. A 20 *μ*m thin gold foil is irradiated with conditions similar to that during the electron angular distribution measurement. A calibrated magnet based electron spectrometer^18^ is kept along the laser axis (Fig. 1). The electron energy spectra, averaged over 50 laser shots, fits two temperatures, 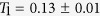 MeV and 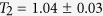 MeV (Fig. 4(c)). For a laser intensity of 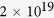 W cm^−2^, the 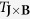 comes to 1.13 MeV which is very close to the fast electron component 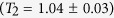 observed in the experiment. This observation once again validates the dominance of **J** × **B** absorption mechanism at relativistic laser intensity.

### Motion of critical surface

It is well established that the absorption mainly occurs near the critical surface, and this surface also gets modified during laser incidence^19^. The motion of critical surface carries an imprint of the laser absorption process^19^. The motion of the critical surface during the laser interaction can be estimated by a launching an external probe to reflect from the critical surface (pump-probe Doppler spectrometry^20^). However, one can also use harmonics generated at the critical surface, to probe the dynamics of the critical surface within the duration of the driving pulse^8^,^21^,^22^. We measure the spectra of the second harmonic emission along the normal at the target front (see method section). Figure 4(d) shows the position of spectral peak of the second harmonic measured during the intensity scan. The dotted horizontal line in Fig. 4(d) shows the location of second harmonic peak estimated from the spectra of the fundamental laser pulse. On the basis of this reference peak, the second harmonic seems to be blue shifted for lower intensities (*I* < 10^17^ W *cm*^−2^), whereas for higher intensities (*I* > 10^17^ W *cm*^−2^) it progressively gets red shifted. Taking the dotted line as a reference spectra, one can estimate the Doppler expansion velocity (*v*), based on the Doppler shift (Δλ), given as *v* = −0.5*c*Δλ/λ, where λ is the wavelength of the second harmonic, and *c* = speed of light in vacuum. The estimated expansion velocity is shown in Fig. 4(d) (violet circles). It is seen that the critical surface is expanding towards the vacuum side at lower intensities, i.e. thermal pressure is dominating over the radiation pressure, whereas for higher intensities, the critical surface is showing an inward motion, i.e. ponderomotive pressure is dominating over thermal pressure^19^,^22^,^23^.

### 2D-PIC simulations

**Figure 5:**
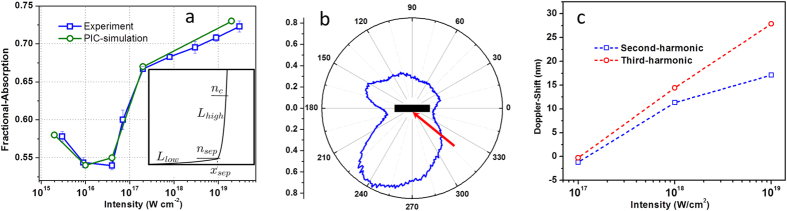
Results from PIC simulation. (**a**) Intensity dependent fractional absorption of simulated ‘*p*’ polarized pulse compared with the experiment. The inset shows the sketch plasma density profile, modelled as two scale lengths (*L*_*low*_ and *L*_*high*_), used in the PIC simulation. (**b**) Simulated angular distribution of fast electrons (*E* > 100 keV). The red arrow and black slab indicates laser direction and target orientation, respectively. (**c**) Simulated intensity dependent spectral shift of the second and third harmonic emission.

Following the experimental results, we try to clarify the involved physical mechanisms using computer simulations. Two-dimensional particle-in-cell (PIC) simulations are performed to verify the experimental measurements related to the data for 10^−9^ intensity contrast. The simulation set up is similar to that in ref. 24, where the *p*-polarized laser at 800 nm is incident from the left boundary of the simulation box at the angle of 40°. The laser intensity profile is given by 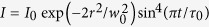, 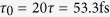 and 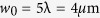. The target is transversely uniform, but with certain longitudinal density profiles. The ion-electron mass ratio of target is set to be 1836 and the initial temperature of both electrons and ions are set as 100 eV. In order to improve the accuracy, we increase the grid resolution and number of macro-particles to 32 cells per laser wavelength and 64 macro-particle per cell, respectively. In the process of trying to reproduce the observed intensity dependent absorption (Fig. 3), we find that a single preplasma scalelength (defined as 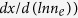 as we used in ref. 24 does not work, i.e., it either fails on the low intensity side or on the high intensity side. This indicates the condition of preplasma is much more complicated in real experiments. Our radiation fluid simulations with MULTI^25^ (results not presented here) show that the preplasma profile are separated into two distinct parts at about 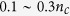 after the interaction with prepulse and ASE^26^. Here 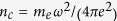 denotes the critical density. Adhering to the target, there is a very thin layer (<1 *μ*m) of plasma with higher density and very short scalelength (~0.1–0.3 *μ*m depending on the incident laser intensity) caused by the thermal expansion of the target material. In front of this layer, a gas density, large-scalelength (>5 *μ*m) preplasma region is generated due to the vaporization, ionization and further expansion of the target surface. Therefore, the preplasma profile in our simulation is set to be 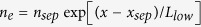 for 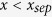 and 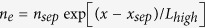 for 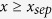 as sketched in the inset of Fig. 5(a), with the intensity-dependent parameters given in Table 1. Using the set up, we finally get the results that are in good agreement with the experiment. Note that for a 40° incidence, the optimal plasma scalelength of resonance absorption^7^ is around 0.2 *μm*. So for the low laser intensity cases, the laser pulse loses little energy when propagating in the low density preplasma part and is mainly absorbed near the critical density due to resonance absorption. However, for high incident intensity, the laser energy is significantly absorbed when it interacts with the low density preplasma by 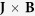 heating and ponderomotive acceleration since these two mechanisms are insensitive to plasma density^27^. We also calculate the angular distribution of the fast electrons and Doppler shift of the second and third-order harmonic of the reflected laser pulse in the simulation, as given in Fig. 5(b,c). There are two dominant emission peaks of hot electrons, one along the incident laser direction and another at the angle between the reflected laser and target normal, as predicted theoretically^28^. At the laser intensity over 10^17^ W cm^−2^, the reflected laser light is red-shifted. The results show good agreement with the experiment.

## Discussion

We have carried out experimental investigations of the absorption of relativistic femtosecond laser pulses by solids over a wide range of laser intensity and laser contrast ratio. In particular, we have (a) changed the laser intensity contrast by four orders of magnitude (10^−5^ −10^−9^), (b) varied laser intensity by four orders of magnitude (10^15^ −10^19^ W cm^−2^), and (c) used four independent, well established diagnostics (reflectivity measurement, angular distribution and energy spectra of fast electrons and Doppler spectrometry of harmonics). The contrast dependent absorption study reveals that an arbitrary increase in the laser contrast cannot lead to higher coupling. For the highest contrast pulse, absorption shows a consistent increasing trend with laser intensity. At 40° of angle of incidence, for high contrast and intermediate intensity, the absorption for ‘*p*’ polarized light could be attributed to vacuum heating, whereas for low contrast it could be dominated by resonance absorption. At high laser intensity, **J** × **B** heating^27^ (which works both for ‘*p*’ and ‘*s*’ polarization) dominates over other absorption mechanism. For instance, at relativistic laser intensity, high absorption is found both for ‘*p*’ and ‘*s*’ polarized lasers. Decreasing absorption with intensity at the intermediate 10^−7^ laser contrast level is rather non-intuitive and seems to be an interesting and important finding, which requires further simulations and experiments for clarity. It may result from the interplay between different absorption mechanisms including resonance absorption, vacuum heating, **J** × **B** heating, ponderomotive force acceleration, and relativistic effect^29^,^30^,^31^. For example, it has been shown that the relativistic effect tends to reduce the resonance absorption rate as the laser intensity increases^32^,^33^. At the highest intensity (10^19^ W cm^−2^), results from all the four diagnostics collectively point towards the dominance of the **J** × **B** mechanism. The experimental results are strongly supported by 2D PIC simulations. One may note that there is apparent discrepancy between the experimental and simulation results in the angular distribution of fast electrons near 240°. There are a few factors which may be responsible for this discrepancy. Firstly, some particular density profile has been taken along the longitudinal direction only, assuming a uniform distribution in the transverse direction in our 2D simulation. In real experiment with 3D geometry, the density distribution is normally not uniform both in the longitudinal and transverse directions in the preplasma. Secondly, a simple Gaussian transverse profile has been taken for the laser pulse, while it may be quite different in our experiment, where the laser has elliptic transverse profile. Finally, the angular distribution found with the peak around 240° from 2D simulation agrees qualitatively with the theory proposed in ref. 28, which suggests that fast electrons will normally eject in an angle smaller than that consistent with the specular reflection of the laser pulse. The findings of this work provide not only a new insight into the physical picture of relativistic intense laser interactions with solid targets, but also a guidance towards the optimization of laser energy coupling to high-energy-density plasmas.

## Methods

The experiment (Fig. 1) is performed at TIFR, Mumbai using 20 TW and 100 TW CPA laser systems (10 Hz). A 30 fs light pulse focused by an f/3.5 off-axis parabola to an elliptical spot (3 *μm* × 10 *μm*), is used to excite the polished bulk aluminium targets at an incident angle of 40°. In order to measure the absorption, the specular reflection from the target is captured by a 3-inch collimating lens kept at a distance of 15 cm from the target and fed into a pyroelectric energy meter. The intensity on the target plane is varied by defocusing the target from the best focal plane. Experiments with 10^−5^ contrast were performed with 20 TW laser system, while using 100 TW laser system, the contrast was varied (from 10^−7^ to 10^−9^) by controlling the energy of seed pulse in the regenerative amplifier. For characterization of the picosecond contrast of the laser pulse, a third-order cross-correlator (SEQUOIA, Amplitude technologies) is used. The contrast trace of fully amplified laser pulse is obtained over several hundreds of laser shots fired at the repetition rate of 10 Hz. A half-wave plate is used to change the polarization of the laser pulse from ‘*p*’ to ‘*s*’. To measure the angular distribution of fast electrons, a curved image plate (Fujifilm BAS-2025), covering a wide angle of nearly 300°, is placed around the 10 *μ*m thick copper foil target. To avoid the low energy radiation of x-rays or electrons hitting the image plate, a 55 *μ*m thick aluminium foil is placed before the image plate. Such a thick Al foil would stop x-rays up to 5 keV, electrons up to 90 keV and protons up to 2.5 MeV. A UV-VIS-NIR spectrometer (Avantes-2048, spectral range 200–1100 nm) is used to capture the emission from the target, as shown in the Fig. 1. In order to avoid the laser light at 800 nm saturating the signal, a Schott BG-39 filter is placed before the spectrometer. The simulations are carried out by our self-encoded 2D3V PIC code KLAPS^34^,^35^. Binary Coulomb collisions between electron-electron, electron-ion and ion-ion have been included in our code using an algorithm based on ref. 36. The collision module is benchmarked according to Landau’s analytical theory of balancing between different temperature component^37^ and collisional absorption^38^. The collision module allows us to include both collisional and collisionless absorption mechanisms self-consistently when calculating the laser absorption over a wide laser intensity range from non-relativistic to highly relativistic.

## Additional Information

**How to cite this article**: Singh, P. K. *et al.* Contrasting levels of absorption of intense femtosecond laser pulses by solids. *Sci. Rep.* **5**, 17870; doi: 10.1038/srep17870 (2015).

## Figures and Tables

**Table 1:** Preplasma scalelength used in simulation for various incident intensities.

Intensity [W·cm^−2^]	*L*_*low*_ [*μ*m]	*L*_*high*_ [*μ*m]	*n*_*sep*_[*n*_*c*_]
2 × 10^15^	0.240	0.152	0.1
1 × 10^16^	1.440	0.192	0.1
4 × 10^16^	1.520	0.188	0.1
2 × 10^17^	2.400	0.184	0.2
2 × 10^19^	10.40	0.400	0.2
